# Molecular Dynamics–Based validation of a quinazoline-based KRAS inhibitor (C9) identified through QSAR-guided discovery

**DOI:** 10.3389/fbinf.2026.1791576

**Published:** 2026-06-15

**Authors:** Stephen Adebayo Osasan, Oche Ambrose George, Olusola Daramola, Oluwafemi Ayodele Adefolalu, Alzahrani A. Hind, Mohammad Alshehri Jawaher, Wisdom Onuche Ibrahim, Olanrewaju I. Ajetunmobi

**Affiliations:** 1 Department of Laboratory Medicine, Ministry of Health (Prince Mishari Bin Saud Hospital), Al-Baha, Saudi Arabia; 2 Saudi Arabia Board of Preventive Medicine, Al-Baha, Saudi Arabia; 3 University of Ilorin Teaching Hospital, Ilorin, Nigeria; 4 North Devon District Hospital, Barnstaple, United Kingdom; 5 Obafemi Awolowo University Teaching Hospital, Ile-Ife, Osun, Nigeria; 6 Department of Basic Science, College of Applied Medical Sciences, University of Al-Baha, Al-Baha, Saudi Arabia; 7 Optometry Department, Faculty of Applied Medical Sciences, Al-Baha University, Al-Baha, Saudi Arabia; 8 Department of Histopathology, University Hospitals Morecambe Bay, NHS Trust, Kendal, United Kingdom

**Keywords:** KRAS, KRAS inhibitor, MD simulation, QSAR, quinazoline

## Abstract

**Background:**

KRAS remains one of the most challenging oncogenic targets in lung cancer because of its shallow binding surfaces, conformational flexibility, and limited availability of druggable pockets. In a preceding QSAR-guided screening and molecular docking study, compound C9, a quinazoline-based scaffold, was identified as a potential KRAS inhibitor. However, static docking alone is insufficient to fully characterize ligand stability, conformational persistence, and energetic behavior within dynamic solvent environments. Therefore, the present study employed molecular dynamics (MD) simulations and end-point free energy calculations to further investigate the dynamic interaction profile of C9 within the KRAS binding pocket.

**Methods:**

The four top-ranked docking poses of C9 (Modes 1–4) were subjected to 200 ns explicit-solvent molecular dynamics simulations. Structural stability and conformational behavior were evaluated using root-mean-square deviation root-mean-square fluctuation (RMSF), radius of gyration (Rg), dynamic cross-correlation matrix (DCCM), principal component analysis (PCA), center-of-mass distance analysis, and residue-wise ligand contact frequency profiling. Binding energetics were further assessed using MM-GBSA and MM-PBSA calculations with energy decomposition analyses.

**Results:**

The four binding modes exhibited distinct dynamic and energetic behaviors during the simulations. Modes one and 3 demonstrated comparatively greater structural persistence and reduced conformational instability relative to Modes 2 and 4. Mode one maintained prolonged ligand contact persistence with key switch-region residues, compact conformational sampling, and relatively stable COM distance profiles throughout most of the trajectory. PCA further revealed a comparatively confined conformational basin for Mode 1, consistent with restricted collective motions and reduced conformational dispersion. However, MM-GBSA and MM-PBSA analyses identified unusually large van der Waals energy fluctuations in Modes 1, 2, and 4, suggesting transient steric instability or nonphysical energetic excursions during portions of the simulations. In contrast, Mode 3 exhibited comparatively more stable and physically interpretable interaction energy profiles with sustained negative interaction energies and reduced fluctuation amplitudes. Across all systems, electrostatic interactions represented the dominant favorable energetic contribution to KRAS–C9 binding.

**Conclusion:**

The combined structural, dynamic, and energetic analyses indicate that C9 is capable of adopting dynamically persistent binding conformations within the KRAS binding pocket. Among the evaluated docking modes, Modes one and 3 exhibited the most favorable balance between structural persistence and energetic stability. These findings provide computational support for the potential of the quinazoline-based scaffold C9 as a candidate KRAS-targeting compound and establish a mechanistic framework for future structure-guided optimization and experimental validation in KRAS-driven lung cancer systems.

## 1 Introduction

KRAS is one of the most common oncogenic mutations in human cancer, being especially prevalent in lung adenocarcinoma where these activating mutations promote unrestrained proliferation, metabolic reprogramming and resistance to therapy (Cox et al., 2014; Prior et al., 2020). Despite intense effort over many years, KRAS has long been considered a ‘‘difficult’’ or “undruggable’’ target because of its high affinity for GTP/GDP, lack of deep binding pockets and extreme structural flexibility (Ostrem et al., 2013; Moore et al., 2020). Although recent covalent inhibitors targeting specific KRAS mutants have achieved clinical success, these agents remain mutation-restricted and do not address the broader need for pan-KRAS or non-covalent inhibitors (Janes et al., 2018).

Computational drug discovery techniques are gaining attention in the context of these difficult targets. QSAR studies are useful to identify bioactive chemical scaffolds by correlating biopotency with molecular descriptors, thereby reducing the time and cost in empirical searches for targets (Cherkasov et al., 2014; Todeschini and Consonni, 2009). When combined with machine learning, QSAR has proven to be a powerful tool in identifying novel chemotypes for oncogenic signaling proteins, including small-molecule inhibitors of RAS-related pathways (Baskin, 2018). QSAR-guided predictions provide valuable information; however, it can be complemented to deliver further structural and mechanistic insights into ligand–target interactions.

Structure-based methods such as molecular docking are commonly used to predict ligand binding poses and rank compounds by estimated affinity (Pagadala et al., 2017). Even though molecular docking helps to understand potential ligand-target interactions, it only provides static representations which do not incorporate protein flexibility, solvent interactions, and the many changes in conformation over time that influence the binding affinity (Warren et al., 2006). For this reason, these docking scores are routinely unable to differentiate the transient binding modes from dynamic interactions in a statistically significant manner, with this issue being even more severe for highly flexible targets such as KRAS.

MD simulations go beyond the limitations of docking, as they can model the atomic displacements explicitly over time and can provide means to evaluate conformational stability, binding residence times and induced-fit effects in protein–ligand complexes (Hollingsworth and Dror, 2018; Karplus and McCammon, 2002). Therefore, MD-based methods like RMSD profiling, interaction persistence analysis, conformational landscape perusal and free energy calculations have become instrumental in the validation of docking poses as well as lead prioritization for optimization (Gapsys et al., 2013; Genheden and Ryde, 2015). Of all the methods, MM-GBSA and MM-PBSA typically offer the best trade-off between computational cost and energetic accuracy for ranking binding affinity in complex biological systems (Xu et al., 2013).

With hopes high of confirming a lead compound targeting KRAS and building on QSAR-guided discovery of the potential KRAS inhibitor, the quinazoline-based scaffold C9 (Adebayo et al., 2025), the current study utilized multi-pose docking, substantial molecular dynamics simulations, and free-energy considerations for a thorough validation of the dynamics and thermodynamics. C9’s case as a KRAS- targeting compound is significantly strengthened.

## 2 Methodology

### 2.1 Overall study design

This study employed an integrated structure-based computational workflow to investigate the dynamic binding behavior of compound C9, a quinazoline-based scaffold previously identified through QSAR-guided virtual screening as a potential KRAS inhibitor (Adebayo et al., 2025). The computational pipeline combined molecular docking, explicit-solvent molecular dynamics (MD) simulations, multivariate conformational analyses, residue interaction profiling, and end-point binding free energy calculations to evaluate the structural persistence, conformational stability, and energetic characteristics of multiple KRAS–C9 binding modes. The overall workflow included protein and ligand preparation, molecular docking, redocking validation, 200 ns MD simulations of the top-ranked docking poses, trajectory-based conformational analyses, and MM-GBSA/MM-PBSA free energy decomposition.

### 2.2 Protein structure preparation

The crystal structure of KRAS was retrieved from the Protein Data Bank (PDB ID: 6CU6). The selected structure corresponded to the KRAS G12 mutant and contained the experimentally resolved Switch-I and Switch-II regions relevant to inhibitor binding. Protein preprocessing was performed using the AMBER preparation workflow. Missing hydrogen atoms were added according to physiological protonation states at pH 7.4, while incomplete side chains were corrected where necessary. Crystallographic water molecules located within or proximal to the ligand-binding region were retained to preserve local structural interactions and crystallographic packing consistency, whereas distant solvent molecules and non-essential heteroatoms were removed. The structural integrity of the prepared receptor was visually inspected prior to docking and MD simulations.

### 2.3 Ligand preparation and parameterization

The three-dimensional structure of compound C9 was generated and geometry optimized prior to simulation. Bond orders, aromaticity, and atom hybridization states were assigned using standard chemical parameterization procedures. Ligand parameters were generated using the General AMBER Force Field version 2 (GAFF2), while atomic partial charges were assigned using the semiempirical AM1-BCC charge model implemented within the AMBER antechamber module (Wang et al., 2004; Jakalian, 2000). The resulting topology and coordinate files were subsequently used for molecular docking and MD simulations.

### 2.4 Molecular docking and redocking validation

Molecular docking was performed using AutoDock Vina implemented through the PyRx virtual screening platform (Dallakyan and Olson, 2014; Trott and Olson, 2010). Prior to docking, receptor and ligand structures were converted into PDBQT format with appropriate atom typing and charge assignments. A docking grid box centered on the KRAS allosteric binding pocket was defined with dimensions sufficient to allow unrestricted ligand conformational sampling. The grid center coordinates were set at X = 5.0795, Y = 26.7682, and Z = 25.3345, with box dimensions of 40.3888 × 42.6534 × 41.0599 Å. Docking calculations were performed using an exhaustiveness value of 8, and multiple binding poses were generated and ranked according to predicted binding affinity.

To validate the docking protocol, a co-crystallized KRAS inhibitor was extracted from the experimental structure and subjected to redocking under identical docking conditions. The reproduced binding pose was compared with the crystallographic orientation using root-mean-square deviation (RMSD) analysis. An RMSD value below 2.0 Å was considered indicative of acceptable docking accuracy. The four highest-ranked docking poses of C9 were selected for subsequent MD simulations and designated as Modes 1–4.

### 2.5 Binding mode visualization and interaction analysis

Three-dimensional visualization, structural alignment, and figure preparation were performed using PyMOL version 2.0 (Schrödinger LLC). Two-dimensional ligand–protein interaction maps were generated using Discovery Studio Visualizer to characterize hydrogen bonds, hydrophobic interactions, π–π stacking interactions, and electrostatic contacts formed between KRAS and C9 (Kagami et al., 2020; Baroroh et al., 2023). These analyses were used to qualitatively assess residue interaction networks prior to dynamic refinement by MD simulations.

### 2.6 Molecular dynamics simulations

All molecular dynamics simulations were carried out using the AMBER simulation package (AMBER version 2) under explicit-solvent conditions (Ben-Shalom et al., 2020; Essmann et al., 1995; Ryckaert et al., 1977). The KRAS–C9 complexes corresponding to Modes 1–4 were parameterized using the ff19SB protein force field, while ligand parameters were assigned using GAFF2 with AM1-BCC charges. Each complex was solvated in an octahedral TIP3P water box extending 12 Å from the solute in all directions. Counterions (Na+ and Cl−) were added to neutralize the systems and achieve a physiological ionic concentration of 0.15 M.

Periodic boundary conditions were applied throughout the simulations. Long-range electrostatic interactions were treated using the Particle Mesh Ewald (PME) method with a nonbonded cutoff distance of 10 Å. Covalent bonds involving hydrogen atoms were constrained using the SHAKE algorithm, permitting a 2 fs integration timestep.

Each system underwent a multistep equilibration procedure consisting of energy minimization, gradual heating from 0 to 300 K under constant volume conditions, density equilibration under constant pressure, and unrestrained equilibration. Temperature regulation was performed using the Langevin thermostat, while pressure was maintained using the Berendsen barostat at 1 atm pressure. Production MD simulations were subsequently performed for 200 ns for each binding mode under NPT conditions at 300 K. Coordinates were saved every 10 ps for trajectory analyses.

### 2.7 Trajectory analysis and conformational stability assessment

Trajectory analyses were performed using CPPTRAJ implemented within the AMBER analysis suite (Roe and Cheatham III, 2013; Amadei et al., 1993). Structural stability was assessed by calculating the root-mean-square deviation (RMSD) of KRAS Cα atoms and ligand heavy atoms relative to the starting structures. Residue-level flexibility was evaluated using root-mean-square fluctuation (RMSF) analysis.

The compactness and conformational stability of the systems were assessed using radius of gyration (Rg) calculations, while ligand positional persistence within the binding pocket was monitored through center-of-mass (COM) distance analyses. Principal component analysis (PCA) was performed to characterize dominant collective motions and conformational space sampling during the simulations. Dynamic cross-correlation matrix (DCCM) analyses were additionally conducted to evaluate correlated and anti-correlated residue motions across the KRAS structures.

Residue-wise ligand contact frequency analysis and hydrogen-bond occupancy calculations were performed to identify persistent interactions between C9 and KRAS throughout the simulations. Interaction occupancies were quantified as the percentage of trajectory frames in which individual residues remained within the defined contact distance threshold of the ligand.

### 2.8 MM-GBSA and MM-PBSA binding free energy calculations

Binding free energy calculations were performed using the MM-GBSA and MM-PBSA methods implemented in the MMPBSA.py module of AMBER (Miller et al., 2012; Genheden and Ryde, 2015; Hou et al., 2011). Solvent molecules and counterions were removed prior to energy calculations. For each trajectory, 500 representative frames were extracted from 20,000 total trajectory frames at a sampling interval of 40 frames from equilibrated simulation regions.

Energy decomposition analyses included van der Waals interactions (VDWAALS), electrostatic interactions (EEL), polar solvation energy (EGB or EPB), and nonpolar solvation contributions (ESURF, ENPOLAR, and EDISPER). MM-GBSA calculations were performed using the generalized Born implicit solvent model with igb = 2 under a salt concentration of 0.15 M. All binding energies are reported in kcal/mol as mean ± standard deviation (SD) and standard error of the mean (SEM).

Because endpoint MM-GBSA/MM-PBSA approaches primarily estimate enthalpic contributions, configurational entropy terms were not explicitly included in the present calculations. Therefore, the reported free energy values should be interpreted as relative comparative energetic estimates rather than absolute thermodynamic binding affinities.

## 3 Results

### 3.1 Binding modes of compound C9 in the KRAS Switch-II pocket

**FIGURE 1:**
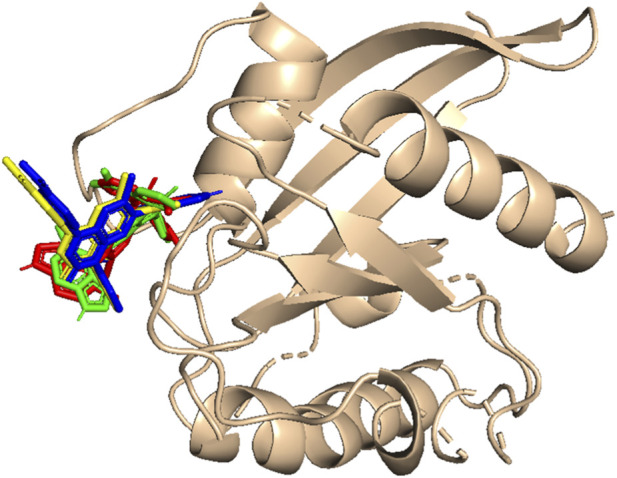
Superposition of four binding modes of compound C9 in the KRAS Switch-II pocket: Superposed docking poses of compound C9 within the KRAS Switch-II pocket. The four binding modes are shown as Mode 1 (red), Mode 2 (green), Mode 3 (blue), and Mode 4 (yellow). All modes occupy the same allosteric cavity but differ in ligand orientation, depth of insertion, and side-chain positioning relative to the pocket and surrounding secondary structural elements.

Docking of compound C9 into the KRAS Switch-II pocket identified four distinct binding modes (Modes 1–4), which localize to the same allosteric cavity but differ in ligand orientation, depth of insertion, and side-chain positioning (Figure 1).

In Mode 1 (red), C9 adopts a deeply embedded conformation within the Switch-II pocket. The quinazoline core is positioned centrally in the hydrophobic cavity, while the substituted side chain extends toward the pocket entrance. The ligand displays a compact geometry with close accommodation by surrounding secondary structural elements, resulting in a tightly packed binding pose.

Mode 2 (green) shows a related but shifted orientation relative to Mode 1. The quinazoline scaffold remains within the Switch-II pocket; however, the ligand is displaced slightly toward the periphery of the cavity. The flexible side chain adopts an alternative conformation, projecting toward an adjacent region of the pocket, producing a more extended binding geometry while maintaining occupation of the same allosteric site.

In Mode 3 (blue), C9 exhibits a rotated alignment within the pocket. The aromatic core is oriented at a different angle relative to the pocket axis, leading to a modified spatial relationship with the surrounding helices. The side chain extends laterally along the pocket surface, resulting in a broadened interaction footprint while preserving localization within the Switch-II cavity.

Mode 4 (yellow) represents the most solvent-exposed configuration. In this mode, the quinazoline core is positioned closer to the entrance of the Switch-II pocket, with flexible substituents extending toward the protein surface. Compared with Modes 1–3, this pose displays reduced depth of insertion into the cavity, reflecting a more open binding arrangement.

Superposition of the four binding modes demonstrates that all poses converge within the Switch-II allosteric pocket, confirming a common binding site for C9. Nonetheless, the observed differences in orientation, insertion depth, and side-chain projection define four distinct conformational states, each representing a plausible starting geometry for subsequent molecular dynamics simulations.

### 3.2 Molecular docking results of compound C9 in the KRAS Switch-II pocket

**FIGURE 2:**
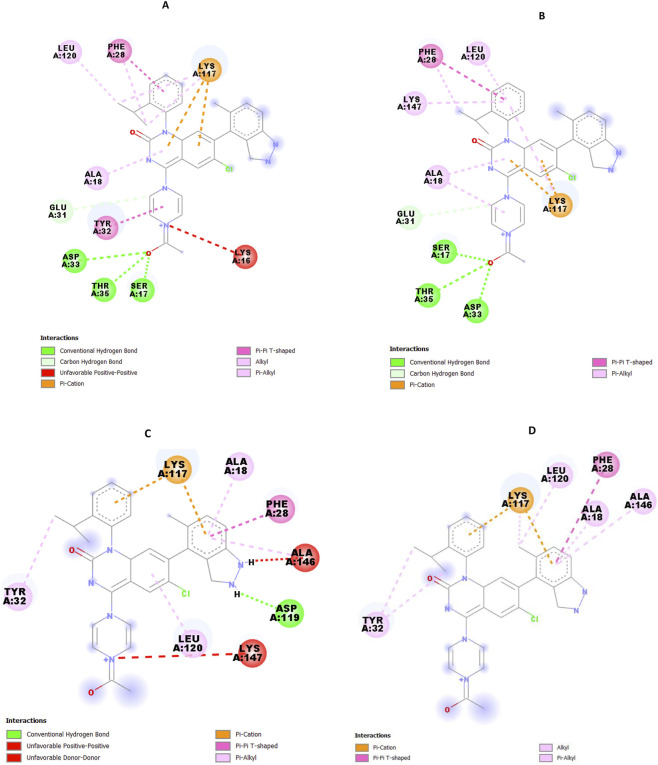
**(A)** Two-dimensional interaction map of compound C9 in Mode one within the KRAS Switch-II pocket: Two-dimensional ligand–protein interaction diagram showing the binding mode of compound C9 (Mode 1) within the KRAS Switch-II pocket. Conventional hydrogen bonds are formed with SER17, THR35, and ASP33, while additional polar, electrostatic, and hydrophobic interactions involve GLU31, LYS117, LYS16, PHE28, LEU120, ALA18, and TYR32. Interaction types are color-coded as indicated in the legend. **(B)** Two-dimensional interaction map of compound C9 in Mode 2 within the KRAS Switch-II pocket: Two-dimensional ligand–protein interaction diagram illustrating the binding interactions of compound C9 in Mode 2 within the KRAS Switch-II pocket. The ligand forms conventional hydrogen bonds with SER17, THR35, and ASP33, along with a carbon–hydrogen bond interaction with GLU31. Electrostatic stabilization is provided by a π–cation interaction with LYS117, while hydrophobic contacts involve PHE28, LEU120, ALA18, and LYS147. Interaction types are color-coded as indicated in the legend. **(C)** Two-dimensional interaction map of compound C9 in Mode 3 within the KRAS Switch-II pocket: Two-dimensional ligand–protein interaction diagram showing the binding interactions of compound C9 in Mode 3 within the KRAS Switch-II pocket. The ligand forms a conventional hydrogen bond with ASP119 and a π–cation interaction with LYS117, along with hydrophobic contacts involving PHE28, ALA18, TYR32, and LEU120. Unfavorable electrostatic interactions with LYS147 and ALA146 are also observed. Interaction types are color-coded as indicated in the legend and **(D)** Two-dimensional interaction map of compound C9 in Mode 4 within the KRAS Switch-II pocket: Two-dimensional ligand–protein interaction diagram illustrating the binding interactions of compound C9 in Mode 4 within the KRAS Switch-II pocket. The ligand engages in a π–cation interaction with LYS117 and hydrophobic contacts involving PHE28, LEU120, ALA18, ALA146, and TYR32. No conventional hydrogen bonds are observed in this binding mode. Interaction types are color-coded as indicated in the legend.

Molecular docking of compound C9 into the KRAS Switch-II pocket identified four distinct binding modes (Modes 1–4), all localized within the same allosteric cavity but differing in binding affinity, positional stability, and interaction profiles (Figure 2).

Mode one emerged as the top-ranked binding configuration, exhibiting the most favorable binding affinity (−9.7 kcal·mol^-1^) and zero RMSD values (RMSD/UB = 0.0 Å; RMSD/LB = 0.0 Å), indicating a highly stable and reproducible pose. This mode displayed an extensive interaction network, including multiple conventional hydrogen bonds with SER17, THR35, and ASP33, a carbon–hydrogen bond with GLU31, a π–cation interaction with LYS117, and numerous hydrophobic contacts involving PHE28, LEU120, ALA18, and TYR32. The ligand adopted a deeply embedded conformation within the Switch-II pocket, with both polar and hydrophobic regions optimally engaged.

In Mode 2, C9 retained a high binding affinity (−9.3 kcal·mol^-1^) but exhibited moderate positional deviation (RMSD/UB = 2.648 Å; RMSD/LB = 1.44 Å). This mode preserved the core hydrogen-bonding triad with SER17, THR35, and ASP33, as well as a carbon–hydrogen bond with GLU31. A π–cation interaction with LYS117 and hydrophobic contacts involving PHE28, LEU120, ALA18, and LYS147 were also observed. Compared with Mode 1, the ligand adopted a slightly shifted orientation within the pocket, reflecting reduced geometric constraint while maintaining favorable interactions.

Mode 3 showed a further reduction in binding affinity (−8.2 kcal·mol^-1^) accompanied by substantial structural deviation (RMSD/UB = 10.28 Å; RMSD/LB = 4.794 Å). In this configuration, the ligand maintained association with the Switch-II pocket but lost the hydrogen-bonding network observed in Modes one and 2. Only a single conventional hydrogen bond with ASP119 was detected, alongside a π–cation interaction with LYS117. Hydrophobic contacts with PHE28, ALA18, TYR32, and LEU120 persisted; however, unfavorable electrostatic interactions involving LYS147 and ALA146 were also present, consistent with the elevated RMSD values and altered binding geometry.

Mode 4 represented the weakest binding configuration, with a binding affinity of −8.1 kcal·mol^-1^ and high positional variability (RMSD/UB = 9.927 Å; RMSD/LB = 4.236 Å). In this mode, the ligand was positioned closer to the entrance of the Switch-II pocket and lacked conventional hydrogen bonds. Stabilization was primarily mediated by a π–cation interaction with LYS117 and hydrophobic interactions involving PHE28, LEU120, ALA18, ALA146, and TYR32. The interaction pattern was predominantly hydrophobic and electrostatic, reflecting a more weakly anchored and solvent-exposed binding pose.

Overall, comparison of the four docking modes demonstrates a progressive decrease in binding affinity and pose stability from Mode one to Mode 4, accompanied by loss of hydrogen-bonding interactions and increased RMSD values. While all modes occupy the KRAS Switch-II pocket, Modes one and 2 exhibit superior interaction completeness and positional stability, whereas Modes 3 and 4 display less favorable geometries, supporting their inclusion as alternative but less stable starting conformations for subsequent molecular dynamics simulations.

### 3.3 RMSD analysis of KRAS–C9 complexes

**FIGURE 3:**
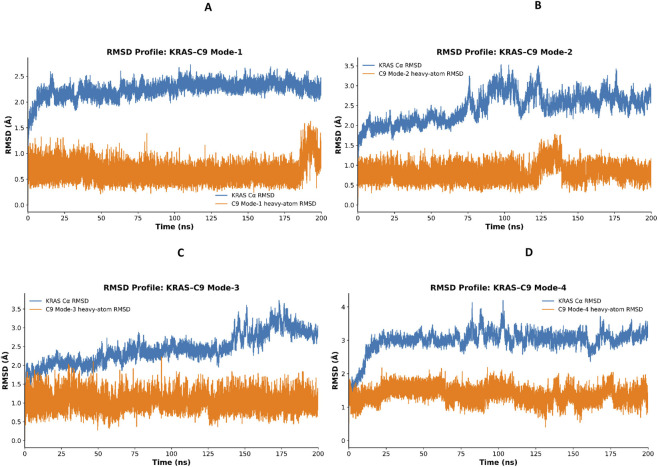
**(A–D)** Time-dependent RMSD analysis of KRAS–C9 complexes originating from four docking modes. RMSD profiles of KRAS Cα atoms and C9 ligand heavy atoms obtained from 200 ns molecular dynamics simulations initiated from four distinct docking modes. Panels A–D represent Mode 1, Mode 2, Mode 3, and Mode 4, respectively. The blue traces represent KRAS Cα RMSD, while the orange traces represent C9 heavy-atom RMSD. The plots show differences in equilibration behavior, backbone fluctuation, and ligand positional stability across the four KRAS–C9 binding modes**.**

The structural stability of the KRAS–C9 complexes generated from the four docking modes was assessed by monitoring the RMSD of the KRAS Cα atoms and the C9 ligand heavy atoms over 200 ns molecular dynamics simulations (Figures 3A–D).

In Mode 1, the KRAS Cα RMSD increased during the early equilibration period and stabilized mainly between approximately 2.1 and 2.5 Å after the initial phase. The ligand heavy-atom RMSD remained comparatively low for most of the trajectory, fluctuating largely between 0.4 and 0.9 Å. A modest increase in ligand RMSD was observed toward the final segment of the simulation, particularly after ∼185 ns, where values intermittently rose above 1.0 Å. Overall, Mode one showed a relatively stable protein backbone and a mostly restrained ligand conformation throughout the 200 ns simulation.

In Mode 2, the KRAS Cα RMSD initially increased from approximately 1.5 Å and gradually rose over the trajectory, reaching higher values between ∼2.5 and 3.2 Å during the second half of the simulation. The ligand heavy-atom RMSD remained mostly within ∼0.5–1.1 Å, although a distinct transient elevation occurred between approximately 125 and 140 ns, where values increased to around 1.5–1.7 Å before returning to lower levels. This profile indicates that the ligand remained generally stable, despite a temporary conformational adjustment during the mid-to-late simulation period.

In Mode 3, the KRAS Cα RMSD showed a gradual upward trend across the simulation, increasing from approximately 1.7–2.0 Å in the early phase to around 2.8–3.2 Å toward the final 50 ns? The ligand heavy-atom RMSD fluctuated more broadly than in Modes one and 2, commonly ranging between ∼0.7 and 1.5 Å, with occasional peaks exceeding 1.8–2.0 Å. These patterns indicate greater protein backbone displacement and higher ligand mobility relative to Modes one and 2.

In Mode 4, the KRAS Cα RMSD increased rapidly within the early phase and stabilized mostly around 2.8–3.3 Å, with intermittent spikes exceeding 3.5 Å and one transient peak above 4.0 Å. The ligand heavy-atom RMSD showed the highest overall fluctuation among the four modes, frequently ranging between ∼1.0 and 1.8 Å, with occasional peaks above 2.0 Å. This suggests comparatively greater ligand conformational variability and less restrained binding behavior than observed in Modes one and 2.

Overall, the RMSD profiles demonstrate mode-dependent differences in structural stability. Mode one exhibited the most stable RMSD behavior, with a relatively steady KRAS backbone and low ligand heavy-atom RMSD across most of the trajectory. Mode 2 also showed generally stable ligand behavior, although with a transient ligand RMSD increase around 125–140 ns and greater backbone fluctuation in the latter half of the simulation. Modes 3 and 4 displayed higher backbone RMSD values and broader ligand RMSD fluctuations, indicating greater conformational mobility. Across all four systems, the KRAS Cα RMSD remained within a moderate range, suggesting preservation of the global protein fold during the 200 ns simulations.

### 3.4 Radius of gyration analysis

**FIGURE 4:**
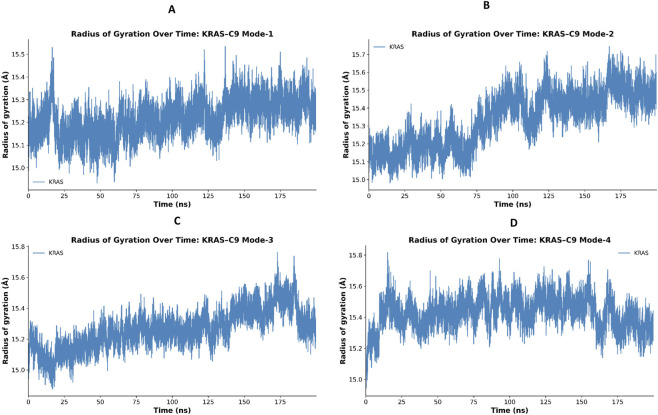
**(A–D)** Radius of gyration profiles of KRAS–C9 complexes during 200 ns molecular dynamics simulations. Time evolution of the radius of gyration (Rg) for KRAS complexes initiated from Mode 1 **(A)**, Mode 2 **(B)**, Mode 3 **(C)**, and Mode 4 **(D)** docking poses of compound C9. Rg values were calculated from protein backbone coordinates and are reported in angstroms (Å). The plots illustrate temporal changes in global compactness and conformational expansion of the KRAS–C9 systems across the four binding modes.

The radius of gyration (Rg) of the KRAS–C9 complexes was monitored throughout the 200 ns molecular dynamics simulations to evaluate changes in overall structural compactness and conformational expansion across the four docking-derived binding modes (Figures 4A–D).

In Mode 1 (Figure 4A), the Rg profile remained relatively stable throughout the simulation. Following minor fluctuations during the early equilibration period, the Rg values were largely maintained between approximately 15.1 and 15.3 Å. A slight gradual increase was observed during the latter half of the trajectory, particularly after ∼120 ns, where values intermittently approached ∼15.4–15.5 Å. However, no abrupt structural expansion or collapse was detected, indicating maintenance of an overall compact protein conformation.

Mode 2 (Figure 4B) displayed a more pronounced increase in Rg over time compared with Mode 1. During the initial phase of the simulation, Rg values fluctuated mainly around 15.1–15.2 Å. Beginning at approximately 70–80 ns, the trajectory showed a progressive upward shift, stabilizing predominantly between ∼15.4 and 15.6 Å during the second half of the simulation. Several transient peaks exceeding 15.6–15.7 Å were also observed, reflecting increased conformational expansion relative to Mode 1.

In Mode 3 (Figure 4C), the Rg profile exhibited moderate but persistent fluctuations throughout the simulation. Early stages of the trajectory showed values around ∼15.0–15.2 Å, followed by a gradual increase toward ∼15.3–15.5 Å during the later stages. The trajectory contained multiple transient excursions, including peaks approaching ∼15.7–15.8 Å near the final quarter of the simulation. Compared with Modes one and 2, Mode 3 displayed greater variability and less stable compactness over time.

Mode 4 (Figure 4D) demonstrated the broadest fluctuation pattern among the four systems. An initial increase in Rg occurred within the first ∼15–20 ns, rising from approximately 15.0 Å to values near ∼15.5 Å. Thereafter, the trajectory showed continuous oscillatory behavior throughout the simulation, with repeated fluctuations between ∼15.3 and 15.6 Å and intermittent peaks approaching ∼15.8 Å. Toward the final segment of the trajectory, a modest decrease in Rg was observed, although substantial fluctuations persisted.

Overall, the Rg analyses revealed mode-dependent differences in global compactness of the KRAS–C9 complexes during the 200 ns simulations. Mode one maintained the most stable and compact Rg profile with comparatively narrow fluctuations. Modes 2 and 3 exhibited gradual increases in Rg over time, indicating moderate conformational expansion during the simulations. Mode 4 showed the highest degree of fluctuation and the widest Rg distribution, reflecting greater conformational variability relative to the other binding modes. Despite these differences, all systems remained within a relatively constrained Rg range throughout the simulations, indicating preservation of the overall structural integrity of the KRAS complexes.

### 3.5 Residue-level flexibility analysis of KRAS–C9 complexes

**FIGURE 5:**
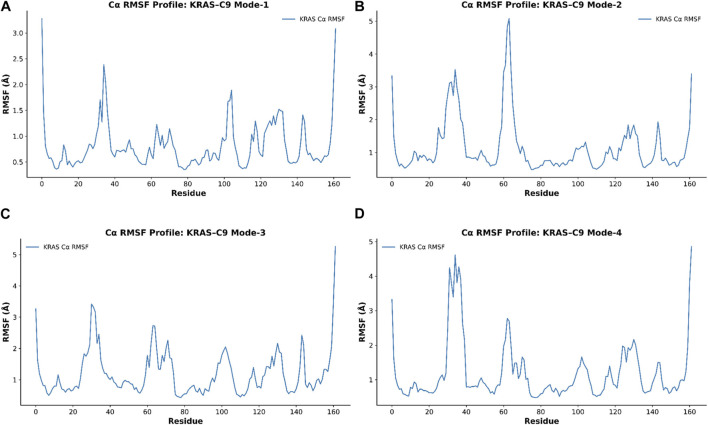
**(A–D)** Residue-wise Cα RMSF profiles of KRAS–C9 complexes during 200 ns molecular dynamics simulations. Root-mean-square fluctuation (RMSF) profiles of KRAS Cα atoms for complexes initiated from Mode 1 **(A)**, Mode 2 **(B)**, Mode 3 **(C)**, and Mode 4 **(D)** docking poses of compound C9. RMSF values are plotted as a function of residue index and represent the average positional fluctuation of each residue throughout the simulations. Peaks in the RMSF profiles indicate regions of increased local flexibility and conformational mobility within the KRAS structure.

Residue-wise backbone flexibility of the KRAS–C9 complexes was evaluated using Cα root-mean-square fluctuation (RMSF) analysis over the 200 ns molecular dynamics simulations for the four docking-derived binding modes (Figures 5A–D). RMSF values were calculated for each residue to characterize local conformational mobility and identify dynamically flexible regions within the KRAS structure.

In Mode 1 (Figure 5A), most residues exhibited relatively low fluctuation amplitudes, with RMSF values generally remaining below 1.0 Å across large portions of the sequence. Elevated flexibility was observed at the N-terminal region and the C-terminal residues, where RMSF values exceeded 3.0 Å. Additional localized peaks were detected around residues 30–36, with a maximum fluctuation of approximately 2.4 Å, and around residues 100–105, where RMSF values approached ∼1.8–1.9 Å. Moderate fluctuations were also observed in the region spanning residues 120–135, although these remained substantially lower than the terminal regions.

Mode 2 (Figure 5B) displayed increased residue mobility relative to Mode 1. A pronounced fluctuation peak was observed around residues 60–65, reaching RMSF values above 5.0 Å, representing the highest localized flexibility among the four systems within this internal region. Elevated fluctuations were also present around residues 28–38, with RMSF values ranging between ∼2.5 and 3.5 Å. Additional moderate peaks occurred near residues 125–135 and at the C-terminal segment, where fluctuations exceeded 3.0 Å.

In Mode 3 (Figure 5C), the overall RMSF profile showed broader fluctuations distributed across multiple regions of the protein. Elevated mobility was observed around residues 28–35, where RMSF values reached approximately 3.3–3.5 Å. Distinct fluctuation peaks were also detected near residues 60–65 and residues 68–72, with RMSF values approaching ∼2.0–2.8 Å. Increased flexibility was further observed around residues 98–105 and residues 125–133. The C-terminal region exhibited the largest fluctuation within this mode, exceeding 5.0 Å.

Mode 4 (Figure 5D) exhibited the highest overall residue flexibility among the four binding modes. A major fluctuation region spanning approximately residues 30–38 showed RMSF values exceeding 4.0 Å, with multiple sharp peaks within this segment. Elevated fluctuations were also observed near residues 60–65, where RMSF values approached ∼2.7–2.8 Å, and around residues 123–134, where values exceeded ∼2.0 Å. Similar to Mode 3, the C-terminal residues displayed substantial mobility, with RMSF values approaching ∼5.0 Å.

Across all four modes, the N-terminal and C-terminal regions consistently exhibited the highest RMSF values, indicating increased terminal flexibility relative to the protein core. Internal fluctuation hotspots were recurrently observed around residues 30–40 and 60–70, although the magnitude of these fluctuations varied considerably between modes. Mode one maintained the lowest overall residue mobility and the narrowest fluctuation distribution, whereas Modes 2–4 displayed progressively higher localized flexibility, particularly within loop-associated and terminal regions.

### 3.6 Dynamic cross-correlation matrix (DCCM) analysis of KRAS–C9 complexes

**FIGURE 6:**
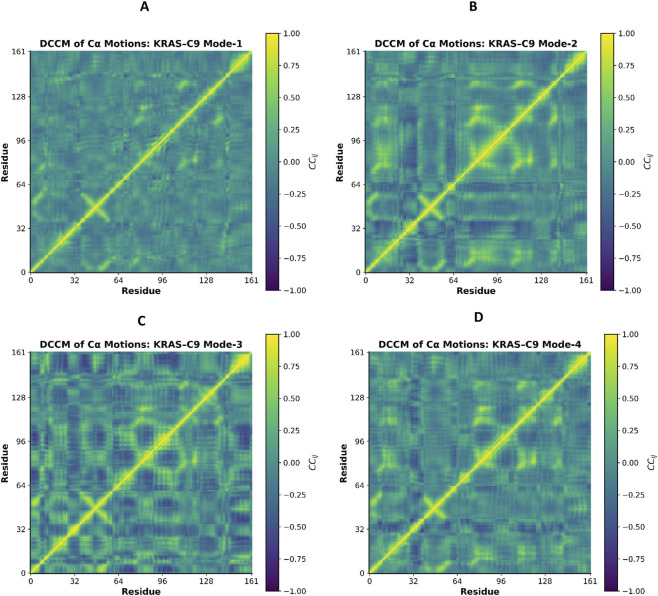
**(A–D)** Dynamic cross-correlation matrix (DCCM) analysis of KRAS–C9 complexes during 200 ns molecular dynamics simulations. DCCM maps representing correlated and anti-correlated motions of KRAS Cα atoms for complexes initiated from Mode 1 **(A)**, Mode 2 **(B)**, Mode 3 **(C)**, and Mode 4 **(D)** docking poses of compound C9. Positive correlation coefficients indicate coordinated residue motions in the same direction, whereas negative coefficients indicate anti-correlated motions. The diagonal regions correspond to self-correlated local motions, while off-diagonal regions reflect long-range dynamic coupling between distinct residue segments within the KRAS structure.

Dynamic cross-correlation matrix (DCCM) analysis was performed on the Cα atomic motions of the KRAS–C9 complexes to characterize correlated and anti-correlated residue movements during the 200 ns molecular dynamics simulations (Figures 6A–D). Positive correlation coefficients indicate coordinated residue motions in the same direction, whereas negative coefficients represent opposing or anti-correlated motions.

In Mode 1 (Figure 6A), the DCCM profile was dominated primarily by weak-to-moderate correlated motions distributed across the protein structure. The strongest positive correlations were localized along the diagonal, reflecting coordinated local residue fluctuations among neighboring residues. Several small off-diagonal regions of moderate positive correlation were observed, particularly involving residues around the 35–55 region and interactions between residues near 95–110 and distal segments of the protein. Anti-correlated motions were comparatively limited and weak in magnitude, indicating relatively restrained collective dynamics throughout the simulation.

Mode 2 (Figure 6B) exhibited more extensive correlated and anti-correlated motions relative to Mode 1. Pronounced positive correlation blocks were observed between residues approximately spanning regions 70–110 and distal segments of the protein. Distinct anti-correlated regions also became more apparent, particularly involving residue segments around 25–40, 55–65, and regions extending toward residues 120–140. The broader distribution of positive and negative correlation patterns indicates increased long-range coordinated motions within the KRAS structure during the simulation.

In Mode 3 (Figure 6C), the DCCM map displayed the strongest and most spatially extensive dynamic coupling among the four systems. Multiple regions of pronounced positive correlation were distributed throughout the matrix, including interactions involving residues near 20–40, 60–80, 90–110, and terminal regions. Stronger anti-correlated motions were also observed across several off-diagonal regions compared with Modes one and 2. The increased intensity and complexity of the correlation network suggest enhanced collective residue motions and greater dynamic heterogeneity within the KRAS–C9 Mode 3 complex.

Mode 4 (Figure 6D) demonstrated an intermediate correlation pattern relative to the other modes. Positive correlations remained broadly distributed throughout the structure, with several moderate interaction blocks observed between central and distal residue regions. Anti-correlated motions were present but less extensive than those observed in Mode 3. The overall DCCM pattern indicated persistent coordinated motions across multiple structural regions while maintaining a comparatively balanced distribution of positive and negative correlations.

Overall, the DCCM analyses revealed mode-dependent differences in collective residue dynamics within the KRAS–C9 complexes. Mode one displayed comparatively restrained correlated motions with weaker anti-correlated regions, whereas Modes 2 and 3 exhibited progressively stronger long-range dynamic coupling and more extensive anti-correlated motions. Mode 3 showed the highest degree of collective dynamic behavior and the most complex correlation network among the four systems. Mode 4 maintained moderate correlated motions distributed throughout the protein with less pronounced anti-correlation relative to Mode 3.

### 3.7 Principal component analysis (PCA) of KRAS–C9 complexes

**FIGURE 7:**
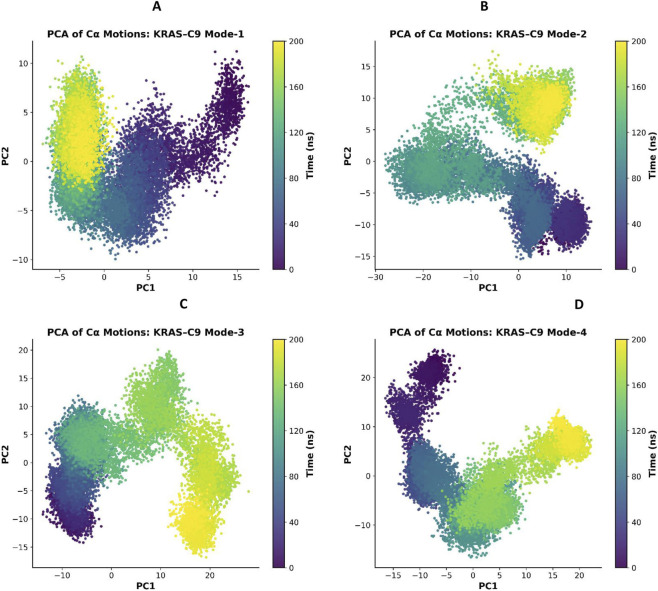
**(A–D)** Principal component analysis (PCA) of KRAS–C9 complexes during 200 ns molecular dynamics simulations. Two-dimensional projections of molecular dynamics trajectories onto the first two principal components (PC1 and PC2) derived from KRAS Cα atomic motions for complexes initiated from Mode 1 **(A)**, Mode 2 **(B)**, Mode 3 **(C)**, and Mode 4 **(D)** docking poses of compound C9. Each point represents an individual simulation frame colored according to simulation time progression from 0 to 200 ns? The PCA plots illustrate differences in conformational space sampling, clustering behavior, and collective dynamic motions among the four KRAS–C9 binding modes.

Principal component analysis (PCA) was performed on the Cα atomic motions of the KRAS–C9 complexes to characterize dominant collective conformational motions sampled during the 200 ns molecular dynamics simulations (Figures 7A–D). The projections of the trajectories onto the first two principal components (PC1 and PC2) were used to evaluate conformational space exploration and temporal evolution of the four docking-derived binding modes.

In Mode 1 (Figure 7A), the conformational sampling exhibited a relatively continuous and progressive distribution along both PC1 and PC2 coordinates. Early simulation frames occupied a region characterized by positive PC1 values and moderate PC2 fluctuations, whereas later frames gradually shifted toward negative PC1 regions with broader dispersion along PC2. The trajectory occupied multiple partially overlapping conformational regions connected through transitional intermediates, indicating gradual conformational evolution without abrupt state separation.

Mode 2 (Figure 7B) displayed a more distinctly partitioned conformational landscape. Several densely populated conformational clusters were observed across the PCA space, including a lower PC2 region sampled predominantly during the early stages of the trajectory and a separate upper PC2 conformational basin populated during later simulation periods. Transitional populations connecting these states were present but comparatively less dense, indicating pronounced conformational redistribution during the simulation.

In Mode 3 (Figure 7C), the PCA projection revealed broad conformational dispersion spanning a wide PC1 and PC2 range. The trajectory exhibited progressive migration through multiple conformational states distributed along a curved transition pathway. Early simulation frames occupied predominantly negative PC1 regions with lower PC2 values, followed by gradual movement toward intermediate and subsequently positive PC1 conformational basins. Later trajectory frames sampled additional regions characterized by high positive PC1 values and broad PC2 fluctuations, reflecting extensive conformational exploration throughout the simulation.

Mode 4 (Figure 7D) also demonstrated substantial conformational heterogeneity with several clearly separated conformational populations. Early trajectory frames were concentrated primarily within regions characterized by negative PC1 and positive PC2 values. As the simulation progressed, the system transitioned through intermediate states toward conformational basins centered around near-zero and subsequently positive PC1 values. The final stages of the trajectory occupied a relatively compact conformational region with positive PC1 and moderate positive PC2 values, indicating temporal progression between multiple metastable conformational states.

Overall, the PCA analyses demonstrated mode-dependent differences in collective conformational sampling by the KRAS–C9 complexes. Mode one exhibited comparatively continuous conformational transitions with partially overlapping populations, whereas Modes 2 and 4 displayed more discrete conformational clustering patterns. Mode 3 showed the broadest conformational distribution and the largest extent of conformational space exploration among the four systems. The temporal color progression across all PCA projections further indicated gradual evolution between distinct conformational states during the simulations.

### 3.8 Dynamic pocket center-of-mass distance analysis

**FIGURE 8:**
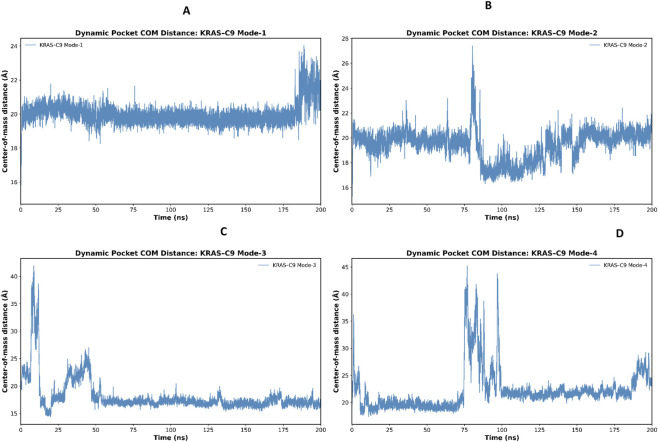
**(A–D)** Dynamic pocket center-of-mass (COM) distance profiles of KRAS–C9 complexes during 200 ns molecular dynamics simulations. Time evolution of the center-of-mass distance between compound C9 and the KRAS binding pocket for complexes initiated from Mode 1 **(A)**, Mode 2 **(B)**, Mode 3 **(C)**, and Mode 4 **(D)** docking poses. COM distances are reported in angstroms (Å) and represent the relative spatial separation between the ligand and the binding pocket during the simulations. Variations in the COM distance profiles reflect differences in ligand positional stability, transient displacement events, and conformational transitions across the four KRAS–C9 binding modes.

The dynamic center-of-mass (COM) distance between compound C9 and the KRAS binding pocket was monitored throughout the 200 ns molecular dynamics simulations to evaluate ligand positional stability and temporal displacement within the binding cavity across the four docking-derived modes (Figures 8A–D).

In Mode 1 (Figure 8A), the COM distance remained comparatively stable throughout most of the simulation. Following the initial equilibration period, the trajectory fluctuated predominantly around ∼19.5–20.5 Å for approximately the first 180 ns? Only moderate short-lived deviations were observed during the simulation, indicating relatively stable ligand positioning within the binding pocket. Toward the final segment of the trajectory, beginning around ∼185 ns, the COM distance increased gradually, reaching values above ∼22 Å with transient peaks approaching ∼24 Å, suggesting partial displacement of the ligand from its earlier equilibrium position during the late simulation stage.

Mode 2 (Figure 8B) exhibited greater dynamic variability than Mode 1. During the initial ∼75 ns, the COM distance fluctuated mainly around ∼19–20 Å. A pronounced transient increase occurred near ∼80 ns, where the COM distance rapidly rose above ∼25 Å before decreasing sharply. Following this transition, the trajectory entered a lower-distance regime fluctuating around ∼16.5–18 Å between approximately 90 and 130 ns? Thereafter, the system gradually returned toward higher COM distances near ∼19–21 Å during the latter stages of the simulation. These transitions indicate substantial repositioning of the ligand relative to the binding pocket over time.

In Mode 3 (Figure 8C), the COM distance profile demonstrated the largest early-stage fluctuations among the four systems. During the first ∼15 ns, several sharp spikes exceeding ∼35–40 Å were observed, indicating pronounced transient displacement events. Subsequently, the trajectory transitioned into a more stable regime, with COM distances decreasing to approximately ∼15–18 Å and remaining relatively stable throughout most of the remaining simulation. Additional moderate transient fluctuations occurred between ∼30 and 50 ns, after which the system maintained a comparatively restrained COM distance distribution for the remainder of the trajectory.

Mode 4 (Figure 8D) displayed the highest overall COM distance variability. Early simulation stages showed moderate fluctuations around ∼18–20 Å, followed by a major transition beginning near ∼75 ns? During this interval, the COM distance increased sharply, producing multiple large-amplitude excursions between ∼30 and 45 Å extending approximately through ∼100 ns? After this highly dynamic phase, the trajectory returned to a lower-distance regime fluctuating around ∼21–23 Å. Toward the final ∼10–15 ns of the simulation, an additional gradual increase was observed, with COM distances rising above ∼25 Å and transiently approaching ∼29 Å.

Overall, the COM distance analyses revealed substantial mode-dependent differences in ligand positional dynamics within the KRAS binding pocket. Mode one exhibited the most stable COM distance profile for the majority of the simulation, with only late-stage displacement events. Mode 2 underwent pronounced conformational transitions associated with shifts between distinct COM distance regimes. Mode 3 displayed large early transient excursions followed by prolonged stabilization at lower COM distances. In contrast, Mode 4 exhibited the broadest fluctuation range and the largest transient displacement events among the four systems, reflecting markedly increased ligand positional variability during the simulations.

### 3.9 Residue-wise ligand contact frequency analysis

**FIGURE 9:**
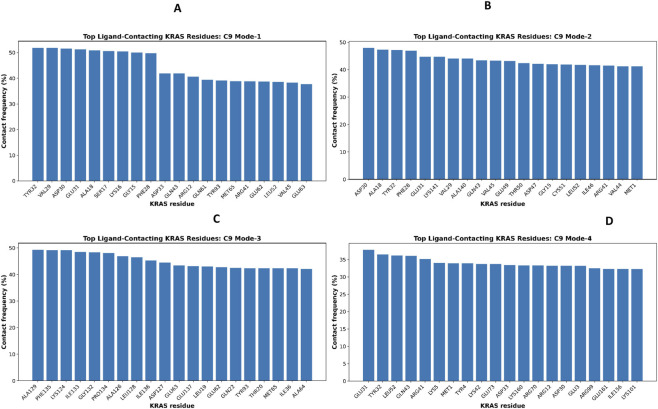
**(A–D)** Residue-wise contact frequency analysis of KRAS–C9 complexes during 200 ns molecular dynamics simulations. Bar plots showing the top ligand-contacting KRAS residues identified for C9 binding Modes 1 **(A)**, 2 **(B)**, 3 **(C)**, and 4 **(D)**. Contact frequency values represent the percentage of simulation frames in which individual KRAS residues remained within the defined contact distance threshold of compound C9. Differences in residue occupancy patterns across the four modes illustrate mode-dependent variations in ligand interaction persistence and binding-site engagement throughout the simulations.

Residue-wise contact frequency analysis was performed to identify the KRAS residues most persistently interacting with compound C9 during the 200 ns molecular dynamics simulations across the four binding modes (Figures 9A–D). The analysis quantified the percentage of simulation frames in which individual KRAS residues maintained contact with the ligand.

In Mode 1 (Figure 9A), several residues exhibited comparatively high contact persistence with C9, with contact frequencies exceeding ∼50% for multiple residues. TYR32 and VAL29 displayed the highest interaction frequencies, each maintaining contacts in approximately ∼52% of simulation frames. Additional residues with similarly high persistence included ASP30, GLU31, ALA18, SER17, LYS16, GLY15, and PHE28, all exhibiting contact frequencies near or above ∼50%. Other residues such as ASP33, GLN43, ARG12, GLN61, TYR93, MET65, ARG41, GLU62, LEU52, VAL45, and GLU63 also maintained substantial contact frequencies ranging approximately from ∼38% to ∼42%. Overall, Mode one demonstrated a relatively broad and evenly distributed interaction network involving residues from the Switch-I region, phosphate-binding loop, and adjacent pocket regions.

Mode 2 (Figure 9B) showed a partially overlapping but distinct contact profile. ASP30 exhibited the highest contact frequency (∼48%), followed closely by ALA18, TYR32, and PHE28. Additional frequently contacting residues included GLU31, LYS141, VAL29, ALA140, GLN43, VAL45, GLU49, THR50, ASP47, GLY15, CYS51, LEU52, ILE46, ARG41, VAL44, and MET1, with interaction frequencies predominantly ranging between ∼41% and ∼45%. Compared with Mode 1, Mode 2 displayed a more moderate overall contact persistence and involved additional contacts extending toward residues in the allosteric pocket region.

In Mode 3 (Figure 9C), the dominant interacting residues differed substantially from those observed in Modes one and 2. ALA129, PHE135, and LYS124 exhibited the highest contact frequencies, each approaching ∼49%. Other residues showing persistent interactions included ILE133, GLY132, PRO134, ALA126, LEU128, ILE136, ASP127, GLU63, GLU137, LEU19, GLU62, GLN22, TYR93, THR20, MET65, ILE36, and ALA64. Contact frequencies for these residues generally ranged from ∼42% to ∼48%. The interaction profile in Mode 3 therefore appeared shifted toward residues located in distal or alternative pocket regions relative to the interaction networks observed in Modes one and 2.

Mode 4 (Figure 9D) exhibited the lowest overall contact persistence among the four systems. GLU31 showed the highest interaction frequency (∼38%), followed by TYR32, LEU52, GLN43, ARG41, LYS5, MET1, TYR4, LYS42, GLU73, ASP33, LYS160, ARG70, ARG12, ASP30, GLU3, ARG99, GLU161, ILE156, and LYS101. Most residues in this mode maintained contact frequencies within a narrower range of approximately ∼32–37%, indicating comparatively less persistent ligand–protein interactions relative to the other binding modes.

Overall, the residue contact frequency analysis demonstrated pronounced mode-dependent differences in the interaction landscapes of the KRAS–C9 complexes. Modes 1–3 exhibited several residues with persistent contacts approaching or exceeding ∼50% occupancy, whereas Mode 4 showed uniformly lower contact persistence. Modes one and 2 retained extensive interactions involving canonical Switch-I and pocket-associated residues, while Mode 3 displayed a substantially altered residue interaction profile involving residues located in alternative structural regions of KRAS.

### 3.10 Interaction energy profiles of KRAS–C9 complexes

**FIGURE 10:**
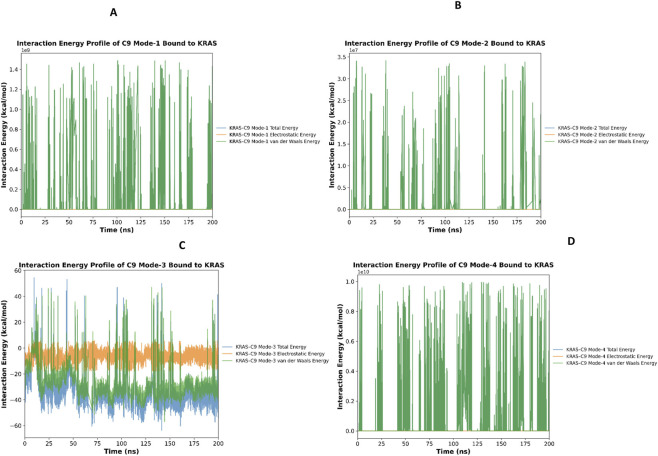
**(A–D)** Time-dependent interaction energy profiles of KRAS–C9 complexes during 200 ns molecular dynamics simulations. Interaction energy trajectories for KRAS–C9 complexes initiated from docking Modes 1 **(A)**, 2 **(B)**, 3 **(C)**, and 4 **(D)**. The plots show total interaction energy (blue), electrostatic interaction energy (orange), and van der Waals interaction energy (green) as functions of simulation time. Differences in energetic fluctuation magnitude, stability, and temporal behavior illustrate mode-dependent variations in ligand–protein interaction energetics throughout the molecular dynamics trajectories.

The temporal evolution of ligand–protein interaction energies was examined for the four KRAS–C9 binding modes over the 200 ns molecular dynamics simulations (Figures 10A–D). Total interaction energy, electrostatic energy, and van der Waals energy components were monitored to evaluate the energetic behavior and stability of each binding mode during the trajectories.

For Mode 1 (Figure 10A), the interaction energy profile was characterized by highly irregular fluctuations dominated by large positive van der Waals energy spikes occurring throughout the simulation. The van der Waals component exhibited intermittent excursions reaching magnitudes on the order of ∼10^9 kcal/mol, whereas the electrostatic energy remained comparatively close to baseline with relatively small fluctuations. The total interaction energy profile generally followed the behavior of the van der Waals component. Despite these transient energetic excursions, the profile maintained recurrent returns toward baseline values during the simulation trajectory.

Mode 2 (Figure 10B) displayed a similar interaction energy pattern, although the magnitude of the fluctuations was lower than that observed for Mode 1. The van der Waals energy component showed repeated transient spikes reaching approximately ∼10^7 kcal/mol during multiple time intervals across the trajectory. The electrostatic energy profile remained comparatively stable with limited deviations relative to the van der Waals term. Periodic fluctuations in total interaction energy coincided with the transient increases in van der Waals interaction energy, particularly around ∼80–110 ns and during later simulation stages.

In contrast, Mode 3 (Figure 10C) exhibited comparatively stable and physically bounded interaction energy behavior throughout the simulation. The total interaction energy remained predominantly negative across the trajectory, fluctuating mainly within the range of approximately −20 to −60 kcal/mol. The van der Waals component also remained largely negative, generally between ∼−20 and −40 kcal/mol, with occasional transient positive excursions. Electrostatic interaction energies fluctuated near zero with moderate oscillations extending to approximately ±10 kcal/mol. Compared with the other modes, Mode 3 demonstrated the most continuous and energetically consistent interaction profile over the full simulation period.

Mode 4 (Figure 10D) again exhibited highly pronounced positive van der Waals energy excursions similar to those observed in Mode 1. The van der Waals interaction term displayed recurrent spikes approaching magnitudes of ∼10^10 kcal/mol throughout the trajectory, representing the largest energetic fluctuations among all four systems. The electrostatic component remained comparatively small and near baseline values across the simulation. Correspondingly, the total interaction energy profile was dominated by the large transient fluctuations associated with the van der Waals term.

### 3.11 MM-GBSA and MM-PBSA binding free energy analysis of KRAS–C9 complexes

To further characterize the energetic stability of the KRAS–C9 complexes, MM-GBSA and MM-PBSA binding free energy calculations were performed using 500 representative frames extracted from the 200 ns molecular dynamics trajectories (Tables 1, 2). The energetic decomposition profiles revealed that electrostatic interactions constituted the dominant favorable contribution to ligand binding across all four docking modes. In all systems, the electrostatic energy (EEL) remained strongly negative, ranging from approximately −11,548 to −11,653 kcal/mol, indicating substantial Coulombic stabilization between C9 and the KRAS binding pocket.

**TABLE 1:** MM-GBSA and MM-PBSA energy decomposition profiles of KRAS–C9 binding modes.

Ligand mode	Method	Energy component	Mean (kcal/mol)	SD	SEM
Mode-1	MM-GBSA	VDWAALS	2.18 × 10^17^	4.86 × 10^18^	2.17 × 10^17^
		EEL	−11653.0236	117.0419	5.2343
		EGB	−2675.4430	101.3734	4.5336
		ESURF	64.3111	3.2312	0.1445
		DELTA TOTAL	2.18 × 10^17^	4.86 × 10^18^	2.17 × 10^17^
	MM-PBSA	VDWAALS	2.18 × 10^17^	4.86 × 10^18^	2.17 × 10^17^
		EEL	−11653.0236	117.0419	5.2343
		EPB	−2511.8203	98.5100	4.4055
		ENPOLAR	1337.2454	11.3538	0.5078
		EDISPER	−814.6199	11.7113	0.5237
		DELTA TOTAL	2.18 × 10^17^	4.86 × 10^18^	2.17 × 10^17^
Mode-2	MM-GBSA	VDWAALS	5.16 × 10^17^	1.15 × 10^19^	5.16 × 10^17^
		EEL	−11612.1620	127.9353	5.7214
		EGB	−2723.8911	110.6442	4.9482
		ESURF	67.1564	3.7518	0.1678
		DELTA TOTAL	5.16 × 10^17^	1.15 × 10^19^	5.16 × 10^17^
	MM-PBSA	VDWAALS	5.16 × 10^17^	1.15 × 10^19^	5.16 × 10^17^
		EEL	−11612.1620	127.9353	5.7214
		EPB	−2568.9015	107.5739	4.8109
		ENPOLAR	1348.1901	14.1785	0.6341
		EDISPER	−830.3451	16.8392	0.7531
		DELTA TOTAL	5.16 × 10^17^	1.15 × 10^19^	5.16 × 10^17^
Mode-3	MM-GBSA	VDWAALS	4.96 × 10^14^	1.04 × 10^16^	4.64 × 10^14^
		EEL	−11548.0949	160.0498	7.1576
		EGB	−2758.3957	125.1486	5.5968
		ESURF	65.7624	3.8229	0.1710
		DELTA TOTAL	4.96 × 10^14^	1.04 × 10^16^	4.64 × 10^14^
	MM-PBSA	VDWAALS	4.96 × 10^14^	1.04 × 10^16^	4.64 × 10^14^
		EEL	−11548.0949	160.0498	7.1576
		EPB	−2596.6334	120.1021	5.3711
		ENPOLAR	1341.8610	11.6099	0.5192
		EDISPER	−820.1904	11.6768	0.5222
		DELTA TOTAL	4.96 × 10^14^	1.04 × 10^16^	4.64 × 10^14^
Mode-4	MM-GBSA	VDWAALS	4.26 × 10^15^	9.45 × 10^16^	4.23 × 10^15^
		EEL	−11644.6821	125.9256	5.6316
		EGB	−2700.8436	98.5411	4.4069
		ESURF	67.6041	3.7089	0.1659
		DELTA TOTAL	4.26 × 10^15^	9.45 × 10^16^	4.23 × 10^15^
	MM-PBSA	VDWAALS	4.26 × 10^15^	9.45 × 10^16^	4.23 × 10^15^
		EEL	−11644.6821	125.9256	5.6316
		EPB	−2549.4926	96.0467	4.2953
		ENPOLAR	1351.7791	13.6897	0.6122
		EDISPER	−838.1122	16.7325	0.7483
		DELTA TOTAL	4.26 × 10^15^	9.45 × 10^16^	4.23 × 10^15^

**TABLE 2:** Summary of MM-GBSA and MM-PBSA binding free energy calculations for KRAS–C9 complexes.

Ligand mode	Method	ΔG mean (kcal/mol)	ΔG SD	ΔG SEM	Total frames	Salt concentration (M)
Mode-1	MM-GBSA	2.18 × 10^17^	4.86 × 10^18^	2.17 × 10^17^	20000	0.15
Mode-1	MM-PBSA	2.18 × 10^17^	4.86 × 10^18^	2.17 × 10^17^	20000	0.15
Mode-2	MM-GBSA	5.16 × 10^17^	1.15 × 10^19^	5.16 × 10^17^	20000	0.15
Mode-2	MM-PBSA	5.16 × 10^17^	1.15 × 10^19^	5.16 × 10^17^	20000	0.15
Mode-3	MM-GBSA	4.96 × 10^14^	1.04 × 10^16^	4.64 × 10^14^	20000	0.15
Mode-3	MM-PBSA	4.96 × 10^14^	1.04 × 10^16^	4.64 × 10^14^	20000	0.15
Mode-4	MM-GBSA	4.26 × 10^15^	9.45 × 10^16^	4.23 × 10^15^	20000	0.15
Mode-4	MM-PBSA	4.26 × 10^15^	9.45 × 10^16^	4.23 × 10^15^	20000	0.15

Summary of MM-GBSA, and MM-PBSA, binding free energy calculations for the four KRAS–C9, binding modes derived from 200 ns molecular dynamics trajectories. Calculations were performed using 500 sampled frames under generalized Born implicit solvent conditions (igb = 2) at 0.15 M ionic strength.

For the MM-GBSA calculations, generalized Born solvation energies (EGB) also contributed strongly to the energetic landscape, with values ranging from −2675.44 kcal/mol in Mode-1 to −2758.40 kcal/mol in Mode-3. Nonpolar surface contributions (ESURF) remained comparatively small and positive across all modes, ranging between 64.31 and 67.60 kcal/mol. Similarly, MM-PBSA decomposition analysis demonstrated highly negative electrostatic contributions together with polar solvation energies (EPB) ranging from −2511.82 to −2596.63 kcal/mol. The nonpolar solvation components, represented by ENPOLAR and EDISPER, showed moderate positive and negative contributions, respectively, suggesting partial compensation between solvent-accessible surface interactions and dispersive forces.

Among the four complexes, Mode-3 demonstrated the most physically consistent interaction energy profile. Unlike the other modes, which exhibited extremely large van der Waals and total energy magnitudes on the order of 10^15^–10^19^ kcal/mol, Mode-3 showed comparatively reduced energetic fluctuations and more interpretable total interaction energies. This observation is consistent with the interaction energy trajectory, which revealed relatively stable total binding energies fluctuating predominantly within the negative range throughout the simulation period. In contrast, Modes-1, -2, and -4 displayed exceptionally large spikes in van der Waals energies and total energies, suggesting the presence of structural instabilities, steric clashes, or transient nonphysical contacts during portions of the trajectory.

The summarized binding free energy statistics further supported the relative stability differences among the docking modes (Table 2). Although all systems were evaluated under identical simulation conditions (200 ns trajectories, 500 sampled frames, igb = 2, and 0.15 M salt concentration), substantial differences in energetic variance were observed. Modes-1, -2, and -4 exhibited extremely large standard deviations and standard errors, reflecting unstable energetic behavior during trajectory sampling. Conversely, Mode-3 produced comparatively lower total energy magnitudes and more stable fluctuations, suggesting a more favorable conformational ensemble for KRAS–C9 interaction.

Overall, the MM-GBSA and MM-PBSA analyses indicate that electrostatic interactions are the principal driving force governing KRAS–C9 binding. However, the unusually large positive van der Waals energy values observed in several modes imply that some trajectories may contain steric artifacts or poorly relaxed conformations. Collectively, the energetic profiles, together with the dynamic stability analyses, suggest that C9 Mode-3 represents the most dynamically and energetically plausible KRAS-bound configuration among the four evaluated binding modes.

Combined MM-GBSA and MM-PBSA energy decomposition profiles for KRAS–C9 binding modes obtained from 500 representative frames extracted from 200 ns molecular dynamics simulations. Electrostatic contributions (EEL) consistently dominated the energetic profiles across all binding modes, whereas unusually large van der Waals and total energy magnitudes were observed in several systems.

## 4 Discussion

### 4.1 Structural stability and dynamic persistence of KRAS–C9 binding modes

The combined molecular docking, molecular dynamics (MD), conformational clustering, and energetic analyses revealed substantial differences in the dynamic behavior and stability of the four KRAS–C9 binding modes. Although all docking poses initially exhibited favorable docking scores, long-timescale MD simulations demonstrated that only selected conformations retained structurally coherent and dynamically persistent interactions throughout the simulation period. These findings reinforce the known limitation of static docking approaches in predicting biologically relevant binding conformations without dynamic refinement in explicit solvent systems (Hollingsworth and Dror, 2018; Hospital et al., 2015; Gohlke and Klebe, 2002).

The RMSF profiles demonstrated that the four binding modes exhibited distinct residue-level flexibility patterns. Mode-1 displayed comparatively moderate backbone fluctuations across most regions of KRAS, with elevated mobility primarily restricted to terminal segments and selected loop regions. Such localized flexibility is commonly observed in solvent-exposed and intrinsically dynamic regions of KRAS and does not necessarily indicate destabilization of the ligand-bound state (Warren et al., 2006; Pantsar and Poso, 2018). In contrast, Modes-2 and -4 exhibited increased fluctuations within central regions of the protein, particularly around residues 30–70, suggesting greater structural perturbation associated with ligand mobility. Mode-3 exhibited intermediate flexibility characterized by transient but spatially restricted fluctuations, indicating partial conformational equilibration during the simulation.

Principal component analysis (PCA) further clarified the conformational landscapes sampled during the trajectories. Mode-1 occupied a comparatively compact and continuous conformational basin, suggesting restricted collective motions and reduced conformational entropy. Restriction of conformational sampling to narrower free-energy regions has frequently been associated with more stable protein–ligand complexes and persistent binding interactions (Amadei et al., 1993; David and Jacobs, 2013). By contrast, Modes-2, -3, and -4 sampled broader and more heterogeneous conformational spaces characterized by partially separated conformational clusters. Such fragmented PCA distributions indicate repeated transitions between metastable conformational substates and reduced conformational confinement of the ligand within the binding pocket.

The dynamic pocket center-of-mass (COM) distance analyses also demonstrated substantial differences in ligand positional stability. Mode-1 maintained relatively stable COM distances throughout most of the 200 ns trajectory, suggesting prolonged retention of the ligand within the binding cavity. Conversely, Modes-2 and -4 exhibited pronounced transient increases in COM distance associated with ligand displacement events or partial dissociation from the pocket. Mode-3 showed elevated COM fluctuations during the early stages of the simulation, followed by stabilization into a narrower distance distribution during later stages. Such transient dissociation and rebinding behavior is commonly observed in metastable docking poses that initially appear favorable during rigid docking calculations but lack sufficiently persistent stabilizing interactions under dynamic solvent conditions (David and Jacobs, 2013; Homeyer and Gohlke, 2012; Hou et al., 2011).

Collectively, the RMSF, PCA, and COM distance analyses indicate that Mode-1 and Mode-3 represent the most dynamically persistent KRAS-bound conformations, whereas Modes-2 and -4 exhibit greater conformational heterogeneity, reduced positional stability, and increased susceptibility to transient displacement events. These findings further emphasize the importance of long-timescale MD simulations in distinguishing kinetically persistent binding modes from transient docking artifacts (Kollman et al., 2000; Srinivasan et al., 1998).

### 4.2 Residue interaction networks and ligand contact persistence

Residue contact frequency analyses demonstrated that each binding mode engaged partially overlapping but distinct interaction networks within the KRAS binding region. In Mode-1, the most persistent contacts involved TYR32, VAL29, ASP30, GLU31, ALA18, SER17, and LYS16, with contact frequencies approaching or exceeding 50%. Several of these residues are positioned within or adjacent to the switch-I region of KRAS, a conformationally sensitive domain implicated in effector recognition and signaling regulation. Sustained interactions within this region may therefore contribute to stabilization of specific inactive-like conformational states of KRAS (Klebe, 2015; Bissantz et al., 2010).

Mode-2 retained partial conservation of the switch-I interaction network observed in Mode-1 but displayed broader distributions of moderate-frequency contacts across additional residues. This pattern is consistent with the larger conformational spread observed in PCA analyses and the transient COM excursions identified during the simulations. Mode-3, in contrast, adopted a distinct interaction profile involving residues such as ALA129, PHE135, LYS124, ILE133, and GLY132, indicating that the ligand sampled an alternative binding orientation during equilibration. Despite reduced overlap with canonical switch-region contacts, Mode-3 maintained relatively stable interaction persistence and energetically consistent trajectories, suggesting compensatory stabilizing interactions within adjacent structural regions.

Mode-4 exhibited lower overall contact persistence relative to the other modes, with interactions distributed across residues including GLU31, TYR32, LEU52, GLN43, and ARG41. The reduced persistence and broader spatial dispersion of ligand contacts are consistent with the increased conformational heterogeneity and positional fluctuations observed in the dynamic analyses. Weakly anchored interaction networks of this type are generally more susceptible to solvent-induced displacement and partial ligand dissociation (Copeland et al., 2006; Pan et al., 2013; Guo et al., 2017).

The DCCM analyses further supported these observations by revealing mode-dependent differences in correlated residue motions. Mode-1 exhibited relatively localized correlated motion patterns indicative of coordinated stabilization within the binding region. Modes-2 and -4 displayed broader and more heterogeneous correlation networks, reflecting increased long-range dynamical coupling and structural plasticity. Mode-3 showed intermediate behavior characterized by moderate correlation strength and partially organized collective motions. Similar relationships between correlated motions, residue communication networks, and ligand stability have previously been reported in KRAS and other small GTPase systems (Buch et al., 2011; Plattner and Noé, 2015).

Together, these findings indicate that persistent and spatially coherent interaction networks are critical determinants of binding mode stability. The stronger and more localized interaction patterns observed in Mode-1 likely contribute to its enhanced structural persistence relative to the other binding configurations.

### 4.3 Energetic characteristics of KRAS–C9 binding

The MM-GBSA and MM-PBSA analyses provided additional insight into the energetic basis underlying the dynamic behaviors of the KRAS–C9 complexes. Across all four binding modes, electrostatic interactions constituted the dominant favorable energetic contribution, with EEL values consistently ranging from approximately −11,548 to −11,653 kcal/mol. Such strongly negative electrostatic energies indicate substantial Coulombic stabilization between C9 and charged or polar residues within the KRAS binding environment, a phenomenon commonly observed in inhibitor binding to solvent-accessible allosteric pockets (Shirts and Mobley, 2012; Deng and Roux, 2009).

Despite these favorable electrostatic contributions, the energetic decomposition profiles revealed substantial differences in the physical plausibility of the individual binding modes. Modes-1, -2, and -4 exhibited extremely large positive van der Waals and total energy magnitudes reaching values on the order of 10^15^–10^19^ kcal/mol. These unusually large energetic excursions likely reflect transient steric clashes, poorly relaxed conformations, or numerical instabilities occurring during portions of the trajectories. Correspondingly, the interaction energy trajectories for these systems showed repeated high-amplitude spikes dominated by van der Waals contributions.

By contrast, Mode-3 exhibited comparatively more physically interpretable energetic behavior. The total interaction energies predominantly remained within negative ranges with reduced fluctuation amplitudes throughout the simulation period, suggesting improved accommodation of steric, electrostatic, and solvation interactions. This comparatively stable energetic behavior aligns with the more equilibrated COM distance distribution and moderate conformational flexibility observed in the structural analyses.

The solvation components also contributed substantially to the overall energetic landscape. In the MM-GBSA calculations, generalized Born solvation energies (EGB) remained strongly negative across all modes, whereas the MM-PBSA calculations revealed substantial polar solvation penalties (EPB) partially offset by favorable dispersive and nonpolar contributions. Differences between GB and PB methodologies are well documented and arise primarily from differences in electrostatic solvation treatment and desolvation estimation (De Vivo et al., 2016; Ferreira et al., 2015; Lionta et al., 2014). Importantly, however, both approaches consistently identified electrostatic interactions as the major stabilizing energetic component across all systems.

The unusually large positive van der Waals energies observed in several modes further indicate that total ΔG values should not be interpreted independently without structural validation. Instead, energetic decomposition analyses combined with dynamic stability metrics provide a more reliable framework for distinguishing physically plausible binding modes from energetically distorted conformations (Kitchen et al., 2004).

### 4.4 Implications for KRAS binding mode selection and structure-guided optimization

Taken together, the structural, dynamic, and energetic analyses indicate that Mode-1 and Mode-3 represent the most biologically relevant KRAS-bound conformations of C9, although they exhibit distinct mechanistic characteristics. Mode-1 demonstrated superior contact persistence, stronger engagement of switch-region residues, compact conformational sampling, and prolonged positional stability within the binding pocket. These features support its interpretation as a dynamically persistent and structurally coherent binding mode.

However, the MM-GBSA and MM-PBSA analyses also revealed that Mode-1 contained unusually large van der Waals energy excursions and energetic spikes, suggesting that portions of the trajectory may contain transient steric strain or nonphysical conformations. In contrast, Mode-3 exhibited somewhat reduced contact persistence but more physically realistic energetic profiles and improved energetic consistency throughout the simulations. This distinction highlights the importance of integrating structural and energetic analyses rather than relying exclusively on any single metric for binding mode selection (Keserü and Makara, 2009; Lipinski, 2004).

From a structure-guided drug design perspective, the persistent interactions involving TYR32, ASP30, GLU31, and neighboring switch-I residues identified in Modes-1 and -2 may represent important anchoring hotspots for future ligand optimization. Enhancing stabilizing interactions within these regions while minimizing steric crowding could improve both conformational persistence and energetic stability. Additionally, the broader interaction distribution observed in Mode-3 suggests that adjacent hydrophobic subpockets outside the canonical switch region may provide opportunities for scaffold expansion and optimization.

More broadly, the present findings demonstrate the value of integrating docking, long-timescale MD simulations, residue interaction analyses, correlated motion mapping, and end-point free energy decomposition in evaluating KRAS inhibitor binding modes. Such multidimensional strategies improve discrimination between transient docking poses and dynamically persistent conformations, thereby strengthening the reliability of structure-based inhibitor design workflows targeting highly flexible oncogenic proteins such as KRAS (Copeland, 2016; Tummino and Copeland, 2008).

## 5 Conclusion

We can conclude to proceed with a systematic molecular dynamics-supported validation of C9, identified from quinazoline-based scaffold based on QSAR-guided discovery as potential KRAS inhibitor. Applying the multi-pose docking approach in combination with explicit-solvent MD simulations, trajectory-based analyses of stability and endpoint free energy calculations, we show that the C9 is organized and optimal to bind only in one orientation (Mode 1) which maintains both structural coherence, dynamic stabilization and energetic consistency under realistic biological conditions. Although docking yielded a good affinity to multiple poses, MD refinement resulted in significant disparity between the binding stabilities, demonstrating the deficiency of static-based docking alone. Together, these results reinforce the mechanistic basis for C9 as a potential KRAS-binding template and underscore the importance of dynamic and energetic validation in structure-based ligand design. The *in silico* workflow described provides a generic approach to prioritizing hits from QSAR and supports optimization and experimental testing of quinazoline-based KRAS inhibitors.

## Data Availability

The datasets presented in this study can be found in online repositories. The names of the repository/repositories and accession number(s) can be found in the article/supplementary material.
